# Correction: Decreased miR-204 in H. pylori-Associated Gastric Cancer Promotes Cancer Cell Proliferation and Invasion by Targeting SOX4

**DOI:** 10.1371/journal.pone.0109057

**Published:** 2014-09-23

**Authors:** The PLOS ONE Staff

There are errors in the Funding section. The correct funding information is as follows: Guoxin Zhang was funded by National Natural Science Foundation of China (No. 81072032 and No. 81270476) and the Priority Academic Program Development of Jiangsu Higher Education Institutions (JX10231801); Xiaoying Zhou was funded by Jiangsu postgraduate scientific research and innovation projects (CXZZ13_0574). The funders had no role in study design, data collection and analysis, decision to publish, or preparation of the manuscript.
